# Are peanut oral food challenges still useful? An evaluation of children with suspected peanut allergy, sensitization to Ara h 2 and controlled asthma

**DOI:** 10.1186/s13223-022-00743-6

**Published:** 2022-11-30

**Authors:** Iida Ojaniemi, Susanna Salmivesi, Antti Tikkakoski, Jussi Karjalainen, Lauri Lehtimäki, Rüdiger Schultz

**Affiliations:** 1 Allergy Centre, Tampere University Hospital, PL 2000, 33521 Tampere, Finland; 2 Faculty of Medicine and Health Technology, Tampere University, Tampere, Finland; 3 Pihlajalinna Medical Centre, Tampere, Finland; 4 Department of Clinical Physiology and Nuclear Medicine, Tampere University Hospital, Tampere, Finland

**Keywords:** Ara h 2, Peanut allergy, Oral food challenge, Asthma, Anaphylaxis

## Abstract

**Background:**

Sensitization to Ara h 2 has been proposed as a promising biological marker for the severity of peanut allergy and may reduce the need for oral food challenges. This study aimed to evaluate whether peanut oral food challenge is still a useful diagnostic tool for children with suspected peanut allergy and an elevated level of Ara h 2-specific IgE. Additionally, we assessed whether well-controlled asthma is an additional risk for severe reactions.

**Methods:**

A retrospective analysis of 107 children with sensitization to Ara h 2-specific IgE (> 0.35 kU/l) undergoing open peanut challenges during 2012–2018 in the Tampere University Hospital Allergy Centre, Finland.

**Results:**

Of the 107 challenges, 82 (77%) were positive. Serum levels of Ara h 2 -sIgE were higher in subjects with a positive challenge than in those who remained negative (median 32.9 (IQR 6.7–99.8) vs. 2.1 (IQR 1.0–4.9) kU/l), p < 0.001) but were not significantly different between subjects with and without anaphylaxis. No correlation was observed between the serum level of Ara h 2-sIgE and reaction severity grading. Well-controlled asthma did not affect the challenge outcome.

**Conclusions:**

Elevated levels of Ara h 2-specific IgE are associated with a positive outcome in peanut challenges but not a reliable predictor of reaction severity. Additionally, well-controlled asthma is not a risk factor for severe reactions in peanut challenges in children with sensitization to Ara h 2.

## Background

Peanut allergy is a steadily growing problem with a global prevalence of 1–2% in Western countries [1, 2]. In 2016, in Tampere, Finland, 2.4% of children starting primary school had physician-diagnosed allergies to either peanut, tree nuts or both [3]. Peanut allergy is often associated with severe reactions, and only approximately 20% of children are outgrowing it [4].

Many of these patients strictly avoid peanuts and are prescribed an epinephrine autoinjector as emergency medication. However, the constant threat of possible exposure and the need for vigilance may have a tremendous negative impact on the quality of life of the patients and their families [5, 6]. This underlines the need for proper diagnostics before starting what will likely be a life-long elimination diet.

Double-blind, placebo-controlled food challenges (DBPCFCs) have been considered the gold standard for the diagnosis of food allergies [7]. Because DBPCFCs are labour intensive for hospitals, patients and families, many centers use open oral food challenges (open OFCs) instead. In any case, food challenges must be performed in specialized centers and with clinicians who are comfortable treating possible severe allergic reactions [8].

Although peanut-specific IgE or skin Prick tests may have a predictive value in the context of a high pretest probability, alone or in combination they are not specific enough for establishing allergy and/or to subside OFCs. Additionally, they do not predict the severity of allergy [9]. Accordingly, the value of other biomarkers have recently been investigated regarding their potential value as substitutes for open OFCs [10]. Sensitization to the component allergen Ara h 2 has been shown to be a good predictor of clinical peanut allergy and it seems to be the best diagnostic test if looking at optimal positive/negative likelihood ratios among presently available testing options [1, 9, 11–13]. Some studies have suggested additionally that Ara h 2-specific IgE could also be a marker for severe peanut allergy [14–16], while other investigators could not establish a connection between Ara h 2-sIgE levels and severity of reaction [12, 17]. However, it is unclear whether there is sufficient evidence that sensitization to Ara h 2 reliably predicts severe reactions in peanut allergic children and could thus be an alternative option to oral food challenges (OFC).

On the other hand, many children with food allergies have asthma as a comorbidity, and coexisting asthma may constitute a considerable risk for generalized reactions in food allergy [18, 19]. Accordingly, to make food challenges as safe as possible, the American Academy of Allergy, Asthma & Immunology (AAAAI) together with the European Academy of Allergy and Clinical Immunology (EAACI) have suggested in their recommendations that possible asthma needs to be well-controlled before performing food challenges [7, 20]. However, there is little evidence regarding whether asthma under optimal control still poses a significant risk for severe allergic reactions while conducting oral food challenges.

The aim of this study was to clarify whether an open challenge test on peanut is still useful and safe for children with suspected peanut allergy and elevated levels of Ara h 2-sIgE. We also wanted to assess whether well-controlled asthma poses an additional risk for severe reactions in Ara h 2 sensitized patients.

## Materials and methods

### Study design and subjects

In the present study, we retrospectively evaluated the data of 107 children with sensitization to Ara h 2 who underwent an open oral food challenge (OFC) to peanut between 2012 and 2018 in the Allergy Centre of the Tampere University Hospital, Finland. Diagnostic serum IgE and/or skin prick tests for sensitization to peanut as well as lung function evaluation to exclude uncontrolled asthma were carried out before performing the OFC. All reactions and medical treatments during the OFC were recorded.

Patient demographics, information about previous allergic symptoms, allergy test results, asthmatic symptoms and the results of lung function tests were collected from the patients’ medical records.

### Tests for allergic sensitization

All patients were evaluated for sensitization to peanut. By the decision of the treating physician and based on their medical history, some patients were additionally evaluated for other allergens, such as tree nuts, aeroallergens and animal dander.

Specific IgE to whole peanut protein and to the heat-stable component Ara h 2 were assessed by using an immunoenzymatic assay (Thermo Fisher Scientific, Uppsala, Sweden) and defined positive when ≥ 0.35 kU/l. Additionally, skin prick tests (SPT) were applied on the child’s forearm using single-head lancets and peanut-specific extract (Tampere University Hospital) with histamine (10 mg/ml) as a positive control and 0.9% saline as a negative control (ALK-Abello, Denmark). Wheal sizes were read after 15 min. SPT was considered positive when the wheal diameter was ≥ 3 mm.

### Lung function tests and asthma

Asthma was diagnosed based on typical symptoms and reversible or variable airflow obstruction according to current guidelines [21–23]. *Current asthma* was defined as having asthma that was treated with regular inhaled corticosteroids, while *ever asthma* also included those subjects who had been diagnosed with and treated for asthma but were currently symptom-free without maintenance asthma medication.

Prior to OFC, probable asthma was assessed according to GINA and ERS guidelines. Additionally, the patients underwent lung function tests to rule out undiagnosed or poorly controlled asthma. For children under 7 years old, impulse oscillometry was applied (Jaeger Viasys, Germany). For children ≥ 7 years, flow-volume spirometry was used (Medikro, Finland). Moreover, challenge tests were used by the decision of the treating physician.

If undiagnosed or poorly controlled asthma was detected, maintenance asthma medication was started or the current asthma medication was adjusted according to current national guidelines for a minimum of 4 weeks to achieve good asthma control before the OFC [21].

### Oral peanut challenge and grading of allergic reactions

OFC was performed using a standardized protocol (Table 1) where the ingested amount of peanut protein was gradually increased within 30-min intervals until the target amount of peanut protein was reached or until either objective or persistent subjective allergic symptoms appeared. Symptoms that justified interruption of the challenge included urticaria, angioedema, vomiting, wheezing or persistent subjective symptoms such as strong abdominal pain. The accumulated amount of ingested whole peanut as well as the amount of peanut protein in milligrams were recorded.

**Table 1:** Open oral food challenge to peanut protocol

	Peanut protein	Whole peanut
Dose 1	2.5 mg	10 mg
Dose 2	25 mg	100 mg
Dose 3	Before October 2015:	
	125 mg for patients < 30 kg	500 mg (1 kernel)
	250 mg for patients ≥ 30 kg	1000 mg (2 kernels)
	After October 2015:	
	250 mg for all patients	1000 mg for all patients

The challenge tests were first categorized as positive and negative based on the judgment of the treating physician. Positive challenge tests were further categorized as anaphylactic/nonanaphylactic according to criteria defined by the EAACI and graded according to their severity by applying Sampson’s criteria [24, 25]. In our study, grades IV–V were classified as severe reactions.

### Statistical analysis

Most of the continuous variables had a nonnormal distribution, and nonparametric tests were used. The Mann–Whitney U test was used to compare continuous variables between two groups, and Spearman’s rho was used to test for correlation. Receiver operating characteristic (ROC) curves were used to test the predictive ability of serum levels of Ara h 2-specific IgE on the outcome of the OFC. The statistical analyses were performed using IBM SPSS Statistics for Windows, version 26 (IBM Corp., Armonk, NY, USA). The results for continuous variables are given as the median (interquartile range, IQR), and a p-value < 0.05 was considered significant.

### Ethics

Patient investigation and clinical work were conducted in compliance with the Declaration of Helsinki. According to local legislations, evaluation by the ethical board is not needed for retrospective chart reviews, and the study was approved as such by the Tampere University Hospital.

## Results

107 Ara h 2-sIgE positive patients underwent OFC with peanut during 2012–2018. Patients were evaluated for possible peanut allergy either due to reported allergic reactions to peanut or sensitization to peanut with no or uncertain exposure. The basic characteristics of the patients are shown in Table 2.

**Table 2:** Subject characteristic

All patients	n = 107
Sex	
Boys	61 (57%)
Girls	46 (43%)
Median age in years, (range)	7.18 (1.17–17.74)
Asthma	
Current	51 (48%)
Ever	60 (56%)
Atopic dermatitis	85 (79%)
Sensitization to any aeroallergen	94 (88%)
Sensitization to Birch (1 missing data)	89 (83%)
Sensitization to tree nuts	94 (88%)
Sensitization to food allergens other than nuts	77 (72%)
Previous history of allergic reactions to peanut	
No known prior reactions	32 (30%)
Suspected prior reactions	75 (70%)
Suspected mild reactions	36 (34%)
Suspected severe reactions	39 (36%)
Median Ara h 2 -sIgE kU/l (IQR)	15.1 (2.8–78.9)
Positive peanut challenge	82 (77%)

A positive oral food challenge was observed in 82 patients (77%), and 41 (50%) of these patients had anaphylaxis according to EAACI’s criteria. Twenty-three patients (28%) could be classified as having a severe reaction of grade IV–V according to Sampson’s criteria [25]. Eighty (98%) children with a positive test outcome received antihistamines, and 38 (46%) received an injection of adrenaline.

### Ara h 2-specific IgE level and reaction severity

**Fig. 1:**
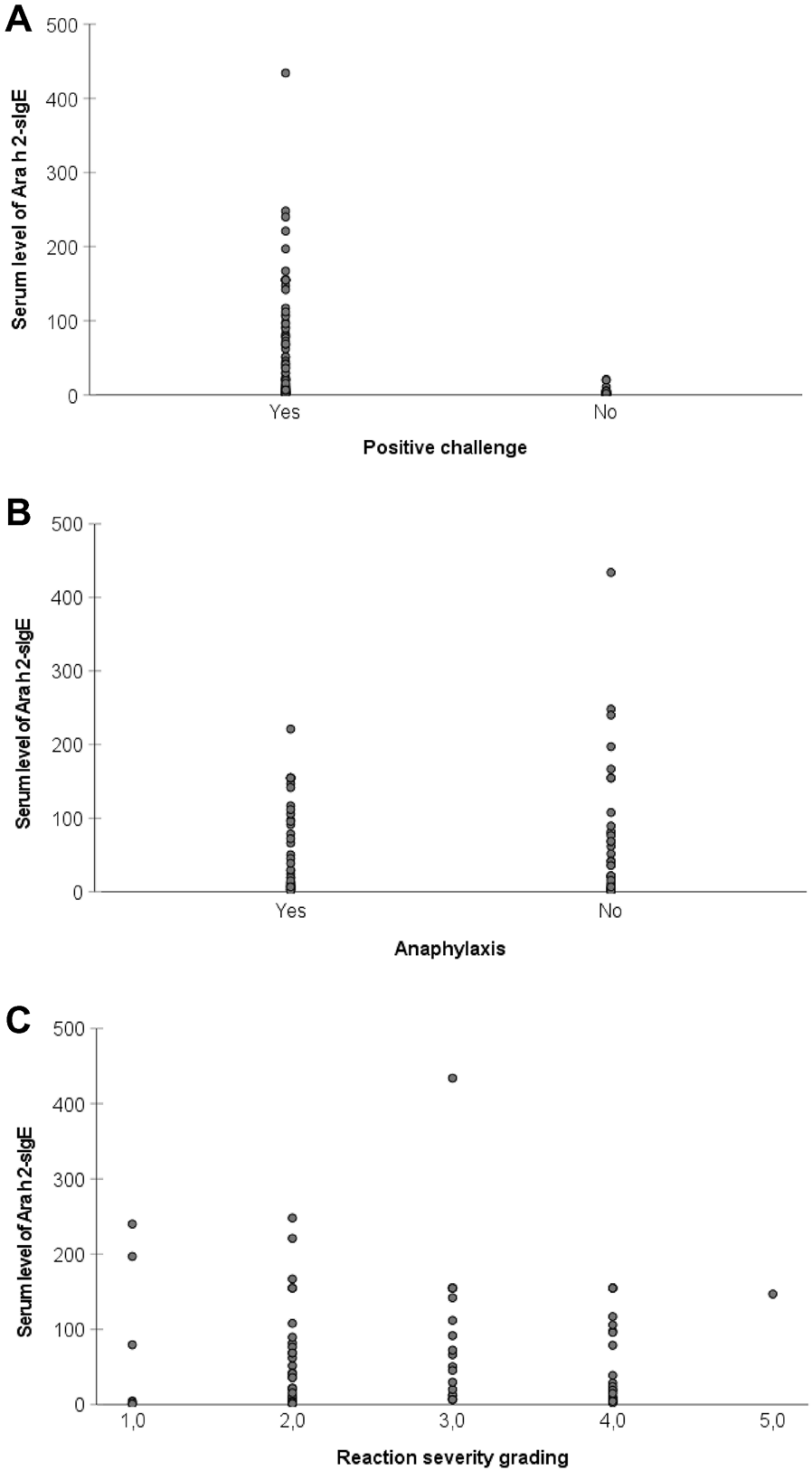
Serum levels of Ara h 2-specific IgE in subjects with positive or negative oral food challenge (**A**), and in subjects with positive OFC according to presence or absence of anaphylaxis (**B**) and according to reaction grading (**C**)

**Fig. 2:**
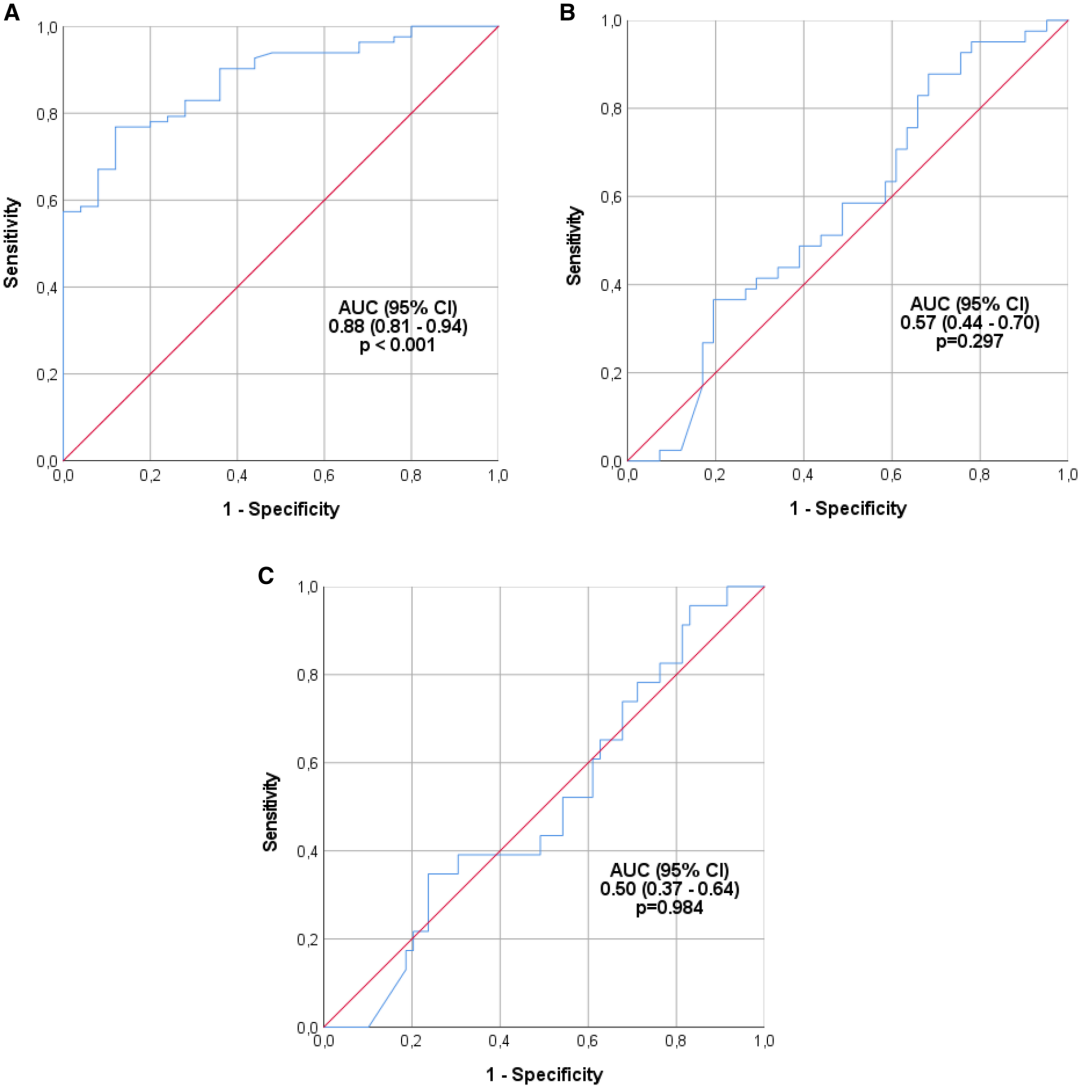
Receivers operating characteristics (ROC) curves of serum Ara h 2-specific IgE when predicting positive outcome (**A**), and anaphylaxis (**B**) or severe reaction (**C**) among positive challenges in oral food challenge test

The serum level of Ara h 2-specific IgE was higher in subjects with a positive OFC than in those with a negative OFC (median 32.9 (IQR 6.7–99.8) vs. 2.1 (1.0–4.9) kU/l, p < 0.001, Fig. 1a), and in the ROC analysis, the area under the curve (AUC) was 0.88 (95% CI 0.81–0.94, p < 0.001, Fig. 2a). OFC was positive in all subjects with a serum level of at least 21.0 kU/l.

In patients with a positive challenge the difference in the serum level of Ara h 2-sIgE was not significant between those who experienced anaphylaxis and those who did not (median 38.9 (IQR 8.8–114.5) vs. 21.9 (3.8–80.7) kU/l, p = 0.297), and in the ROC analysis, the AUC was 0.57 (95% CI 0.44–0.70, p = 0.297, Fig. 2b). When positive challenges were classified into severe (grade IV–V) and nonsevere ones (grade I–III), there was no significant difference in the level of Ara h 2-specific IgE (median 24.1 (IQR 7.3–106.0) vs. 36.4 (IQR 6.6–91.7) kU/l, p = 0.984). In the ROC analysis, the AUC was 0.50 (95% CI 0.37–0.64, p = 0.984, Fig. 2c). We did not observe a correlation between Ara h 2-sIgE level and reaction severity grading (Spearman rho 0.102, p = 0.360). Based on the ROC analysis, there were no clinically useful cutoff levels of Ara h 2-sIgE for predicting reaction severity among those getting allergic reactions in OFCs.

Thirty-two children (30%) had no known previous allergic reactions to peanut. When we performed the same tests on a subgroup of patients (N = 75) who had a history of a suspected allergic reaction to peanut the results remained similar: 60 (80%) challenges were positive. The serum level of Ara h 2-specific IgE was higher in subjects with a positive OFC than in those with a negative OFC (median 36.2 (IQR 8.3–107.6) vs. 2.6 (1.7–5.6) kU/l, p < 0.001, and in the ROC analysis, the area under the curve (AUC) was 0.88 (95% CI 0.80–0.96, p < 0.001). The difference in the serum level of Ara h 2-sIgE was not significant between those who experienced anaphylaxis and those who did not (median 44.7 (IQR 9.6–124.5) vs. 28.9 (4.8–77.9) kU/l, p = 0.403), and in the ROC analysis, the AUC was 0.56 (95% CI 0.44–0.70, p = 0.403). We did not observe a correlation between Ara h 2-sIgE level and reaction severity grading (Spearman rho 0.098, p = 0.456). When positive reactions were analysed by grouping outcomes into severe (grade IV–V) and nonsevere reactions (grade I–III), no significant difference in the levels of Ara h 2-sIgE was found (median 38.9 (IQR 10.0–117.0) vs. 35.9 (IQR 7.5–99.9) kU/l, p = 0.633), and in the ROC analysis the AUC was 0.54 (95% CI 0.38–0.0.69, p = 0.634).

### Asthma and severity of allergic reactions

**Fig. 3:**
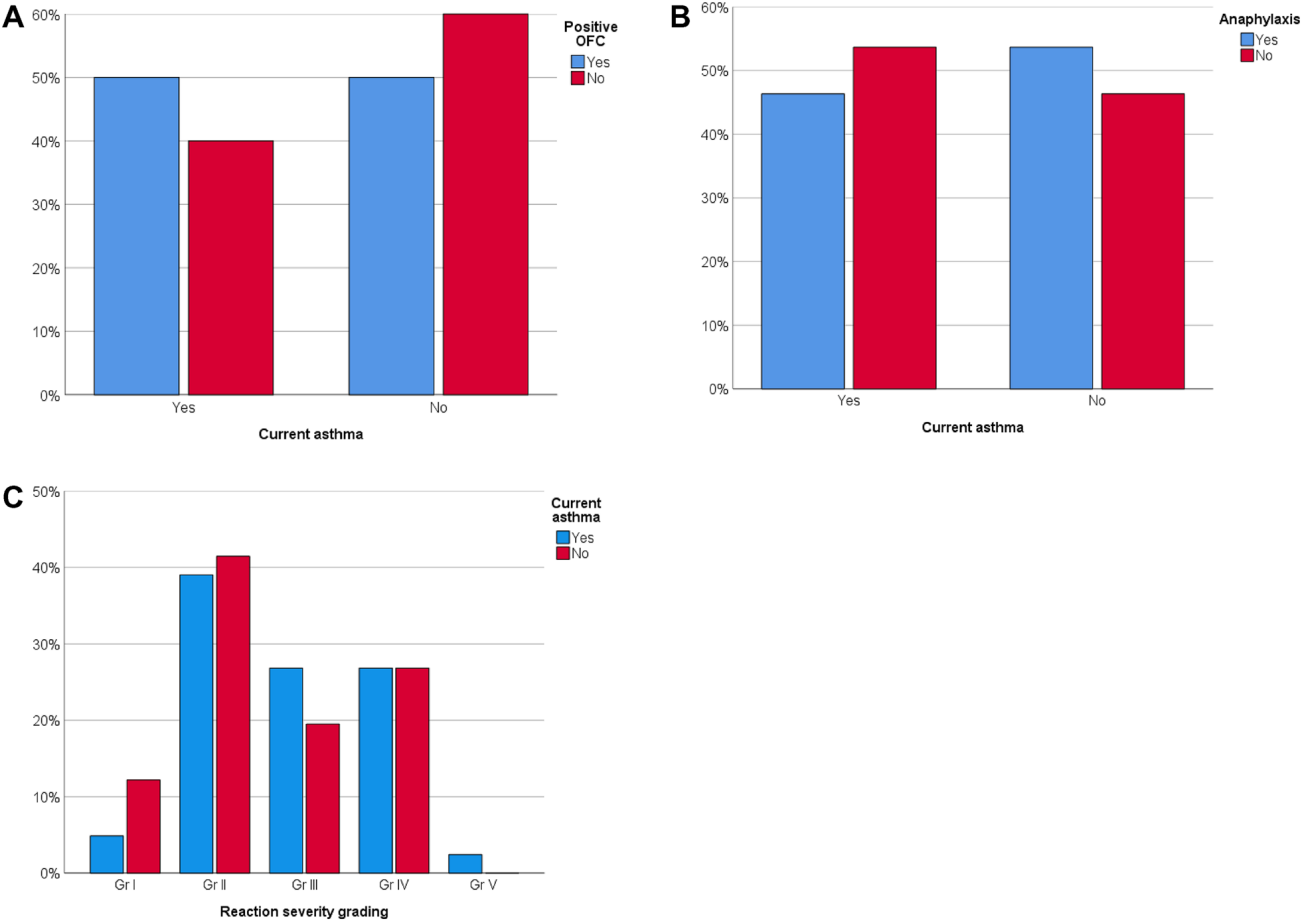
**A** Positive and negative food challenge outcomes among patients with and without current asthma. **B** Anaphylaxis and no anaphylaxis in positive peanut challenges among patients with and without current asthma. **C** Reaction severity grading in positive peanut challenges among patients with and without current asthma

Between subjects with and without current asthma, there were no differences in the proportions of subjects with positive OFCs (80% vs. 74%, p = 0.381, Fig. 3a).

Among positive challenges, the proportions of subjects with and without anaphylaxis were 46% in asthmatic patients and 54% in nonasthmatic patients (p = 0.508, Fig. 3b).

There was no statistically significant difference in the median reaction severity between subjects with and without asthma among patients with positive OFC (gr 3 (IQR 2–4) vs. gr 2 (IQR 2–4), p = 0.354).

## Discussion

In this study, we collected data from 107 children with an elevated serum IgE level of the peanut component Ara h 2 undergoing peanut OFC. The objective of the study was to evaluate the value of Ara h 2-sIgE in predicting severe reactions during OFC and thereby to ponder its clinical value as an alternative to open challenges. Additionally, we wanted to shed light on the question whether controlled asthma constitutes a risk for severe reactions during peanut OFC in Ara h 2-IgE positive patients, which to the best of our knowledge has not been clarified in previous studies.

Concerning the value of Ara h 2-sIgE in predicting severe reactions during peanut challenges, there have been published conflicting results. However, Ara h 2-sIgE has been shown to be a good predictor of peanut allergy in general and can help to distinguish between clinical peanut allergy and sensitization [26]. In our study cohort, all patients were sensitized to Ara h 2 (Ara h 2-sIgE ≥ 0.35 kU/l). Some of these patients had no known prior exposure to peanut and some had been suspected of having experienced allergic reactions due to ingestion of peanut.

The results of this study clearly show that higher levels of Ara h 2-specific IgE were linked to a positive outcome in OFCs but did not predict anaphylaxis or severe reactions (Fig. 1a–c). This is in line with previous studies by van Erp et al. and Errer et al., who obtained similar results [11, 26]. Additionally, we were not able to determine a statistically significant cutoff point for Ara h 2-sIgE levels predicting a severe outcome in peanut OFCs. However, the OFC was positive in all subjects with a serum level of Ara h 2-sIgE ≥ 21.0 kU/l.

Additionally, 23% of our patients with elevated Ara h 2-sIgE levels (range 0.36–20.8 kU/l) had a negative outcome, showing that an Ara h 2-sensitized patient can also be tolerant to peanut. On the other hand, four patients with suspected previous severe allergic reactions to peanut had a negative outcome in the OFC, which underlines the need for confirming the diagnosis of peanut allergy before setting a patient on a life-long elimination diet. Thus, conducting an OFC in patients with elevated Ara h 2-sIgE levels can provide important and relevant information. Those who turn out to be negative in the OFCs are liberated from the fear of allergic reactions and are able to reintroduce peanuts to their diet being advised to start with small amounts of peanut. Even for patients with clinical allergy, an OFC can shed light on allergy severity. OFCs have additionally been shown to improve allergy-related quality of life regardless of the outcome [27].

Underlying asthma in children with food allergies has been proposed to be connected with considerable morbidity and even a fatal outcome in allergic reactions [28–30], but contradictory results have also been reported [11, 31]. Current recommendations propose asthma to be a risk factor for more severe reactions regardless of asthma severity. As recommended, all our patients were evaluated for undiagnosed or poorly controlled asthma according to national and GINA guidelines before conducting the challenge, and if needed, their treatment was optimized [21, 22]. Evaluating the results of our study, even among this Ara h 2 sensitized cohort, we did not find a connection between well-controlled asthma and reaction severity during peanut OFC or even the outcome in terms of positive and negative. Our results are in line with a recent review that found no evidence for asthma being a risk factor for severe reactions if asthma control was satisfactory [32]. Our findings are also supported by previous reports from Petterson et al. and van Erp et al., who reported that asthma in general was not related to reaction severity in OFCs [11, 31]. It seems to be therefore justified and safe to conduct peanut OFC for patients with asthma and elevated levels of Ara h 2-sIgE if their asthma is well-controlled.

In our challenge protocol the dosing and the cumulative amount of peanut protein was smaller compared to some other studies and protocols which might pose the question if some of our patients would have reacted if given a higher dosing of peanut [8]. However, in general, the reported eliciting doses have been still significantly lower than the final dosing in our challenge protocol [8, 33]. Taking also into account the high pretest probability of clinical allergy with all patients having been sensitized to Ara h 2, we believe that our dosing was sufficient enough to trigger a possible reaction.

As shown in recent studies, the addition of the component Ara h 6 could possibly increase the reliability of component-based diagnostics for the prediction of severe reactions in peanut challenges [14, 34]. However, laboratory tests for Ara h 6-sIgE were not available when children were investigated for peanut allergy and therefore Ara h 6-sIgE could not be included into the data pool of this study. To investigate whether Ara h 6-sIgE either alone or in combination with Ara h 2-sIgE may serve as a predictive diagnostic tool for severe reactions during peanut challenges in children a separate, prospective study should be initialized.

## Conclusion

In conclusion, Ara h 2-specific IgE levels may be associated with a positive outcome in peanut challenges but are not a reliable predictor of anaphylaxis or reaction severity. We also found that well-controlled asthma constitutes no additional risk for severe reactions in peanut challenges in children with suspected allergy and sensitization to Ara h 2. Therefore, we suggest that these children should be challenged using standard protocols to prevent unnecessary lifelong elimination diets.

## Data Availability

The datasets used and/or analysed during the current study are available from the corresponding author on reasonable request.

## Abbreviations

OFC: Oral food challenges
DBPCFC: Double-blind, placebo-controlled food challenge
sIgE: Specific IgE
SPT: Skin prick tests
ROC curves: Receiver operating characteristic curves
AUC: Area under the curve
IQR: Interquartile range
CI: Confidence interval
